# Pandemic vaccine testing: Combining conventional and challenge studies

**DOI:** 10.1002/pds.5429

**Published:** 2022-03-31

**Authors:** Tobias Gerhard, Brian L. Strom, Nir Eyal

**Affiliations:** ^1^ Center for Pharmacoepidemiology and Treatment Science Rutgers University New Brunswick New Jersey USA; ^2^ Ernest Mario School of Pharmacy Rutgers University Piscataway New Jersey USA; ^3^ Rutgers Biomedical and Health Sciences Newark New Jersey USA; ^4^ Center for Population‐Level Bioethics Rutgers Biomedical and Health Sciences New Brunswick New Jersey USA; ^5^ Department of Philosophy Rutgers University New Brunswick New Jersey USA; ^6^ Department of Health Behavior, Society and Policy Rutgers School of Public Health Piscataway New Jersey USA

**Keywords:** adaptive clinical trials as topic, coronavirus, ethics, randomized controlled trials as topic, vaccines

## Abstract

Early into COVID, human challenge trials were considered, but usually as alternatives to conventional randomized controlled trials. Instead, assessment of authorized COVID vaccines, of further COVID vaccines, and of vaccines against future pandemics should combine both designs, in five different ways, including a wholly novel one that we elaborate, Viz., combining data from both designs to answer a single question.

## 1 THE NEED FOR CONTINUED VACCINE TESTING

Though multiple SARS‐CoV‐2 vaccines are authorized or approved, further testing could help to settle many open questions—how much they block infection and infectiousness by old and new viral strains long after first administration, which dosing and timing regimens are ideal, what are the correlates of vaccine protection, how each compared to natural infection, and how well they work in populations underrepresented among recruits or infection cases in earlier field trials. There also remains enormous value to testing next‐generation vaccines, ranging from strain‐specific vaccines through universal COVID vaccines to other COVID vaccines that prove even more efficacious at blocking transmission (against some strains), even safer, easier to deliver in resource‐poor settings, cheaper, monopoly‐breaking, or simply possible to manufacture and distribute to uncovered global populations without undermining manufacture of authorized vaccines.

Yet, now that vaccines are increasingly available to more and more residents of rich countries, it is ethically harder to conduct conventional placebo‐controlled trials there.^1^, ^2^, ^3^ Conventional trials in countries whose populations lack vaccine access would also be ethically contentious,^2^, ^4^ or practically impossible.^5^ While a few altruists who give truly informed consent to take on risks may be found around many sites, finding tens of thousands around a single site is unrealistic.

The UK has completed COVID challenge trials and starting others,^6^, ^7^ though those do not primarily assess vaccine efficacy. In a standard COVID *vaccine* challenge trial, a few dozen consenting study volunteers in an isolated medical facility are randomized to receive either the vaccine candidate(s) or control, say, a placebo. After the time period required for development of immunity, all are deliberately exposed to SARS‐CoV‐2. Within weeks, large differences in infection rates, viremia, nasal titer, and other outcomes between the two arms would confirm efficacy in blocking infection and infectiousness, and absence of significant differences would confirm inefficacy. The correlates and duration of vaccine protection and infection kinetics are easily discernible. To minimize risk to challenge trial participants, all current plans suggest recruiting only consenting young adults, free from major risk factors for severe COVID‐19 following SARS‐CoV‐2 infection.^8^, ^9^, ^10^, ^11^

In the 2020 public debate over challenge trials, proponents and opponents alike evaluated them primarily as alternatives to conventional Phase 3 field trials.^8^, ^10^, ^11^, ^12^, ^13^, ^14^, ^15^, ^16^ Indeed, some were puzzled that the United Kingdom is launching challenge trials after conventional trials have completed successfully.^17^ This commentary concerns circumstances when either design would otherwise be permitted as a standalone (an assumption that in our views obtained^8^, ^18^, ^19^ and will continue to obtain^20^ in COVID); it argues that in such circumstances, both now and in some future pandemics, it may be even more advisable to combine conventional and challenge testing for surer, faster, and more comprehensive vaccine assessments and fuller understanding of the infection and the disease.

## 2 A PARALLEL APPROACH

We propose a parallel approach, which runs both a conventional trial and a challenge trial. We call it “Combining Conventional and Challenge trials”—CCC for short. CCC permits either the challenge or the convetional trial to start (or end) before the other (by “parallel” we do not mean *synchronous*). It also permits simultaneous or later observational and other studies.

Assessing each vaccine with both conventional and challenge trials would be advisable in five different ways. First, it would increase the chance that at least one trial works out on its own. Conventional trials can fail to reach prespecified event numbers expediently enough, for example, if population incidence around study sites drops markedly due to more comprehensive mitigation measures. That can happen even when global incidence is high and increasing, and it has happened in some COVID vaccine^21^ and treatment trials.^22^ Morbidity and mortality on a pandemic scale is so vast that expediting development of more effective pandemic response tends to have extremely high social value, which justifies erring on the side of modest redundancy. Challenge trials, likewise, can fail, for example, when a long dose escalation process fails to identify a virus dose that both infects enough participants and remains acceptably safe.

Second, if both trials work out, regulators and the public receive information from more than one trial, which would increase their confidence,^23^ as may have been the case for the typhoid conjugate vaccine.^24^

Third, fast challenge trial results could help select which of many vaccine candidates should advance to a conventional trial, conserving finite resources.^10^, ^25^, ^26^, ^27^

Fourth, these different designs answer different questions about vaccines, per each design's respective scientific strengths.^23^ For example, discerning the impact of vaccines on infection and shedding ratios, dosing, timing, and natural history, and on the correlates of vaccine protection, is easier to do in challenge trials than in conventional trials.^10^, ^11^, ^27^, ^28^, ^29^ However, conventional trials of sufficient size are superior in providing data on vaccines' effects on disease, severe disease, and common severe adverse effects from either toxicity^30^ or possibly from severity enhancement.^31^, ^32^

Finally, and perhaps most intriguingly, one may be able to use data points from both trial types to answer one and the same question about a vaccine that could not be answered by either trial alone. The rest of this analysis explains how that might be done.

## 3 COMBINING DATA FROM CONVENTIONAL AND CHALLENGE TRIALS TO ANSWER A SINGLE QUESTION

Consider a current difficulty. Any new conventional, placebo‐controlled trials are likely to take place in countries with little or no vaccine access.^1^, ^2^ By the time all their participants are recruited, consented, vaccinated, and can develop and demonstrate immunity (altogether, months after the start of the trial), the exposure incidence rate may wane in the trial sites, even if it was very high at its start. Indeed, trialists' publicized predictions of high transmission (based on, e.g., pandemic modeling)^33^ may prompt individuals and governments to intensify mitigation efforts around sites, reducing spread there. That systematically limits trialists' ability to predict levels of community spread around sites. In addition, if the trial lasts long enough, vaccines may become available locally, prompting some placebo arm participants to drop out of trials and making it ethically harder to ask others to stay.^2^, ^3^, ^4^ So in any future COVID conventional vaccine trials, failure to reach ample case accrual for statistically valid results within an acceptable timeframe unfortunately remains a real possibility. All the more so in any noninferiority conventional trial, given the larger sample size needed and the high efficacy of authorized vaccines.

Similar problems may affect trials seeking to tease apart vaccine efficacy against different viral strains. The prevalence of a strain among infection cases (and, among them, among likely infectiousness cases) can only be confirmed after data collection. Only then would exposure to some strains be seen to fall short of statistical significance. Under‐accrual within an acceptable timeframe could also arise in a globally waning pandemic,^11^ and potentially in trials for future emerging infections with more modest spread than SARS‐CoV‐2.

**FIGURE 1:**
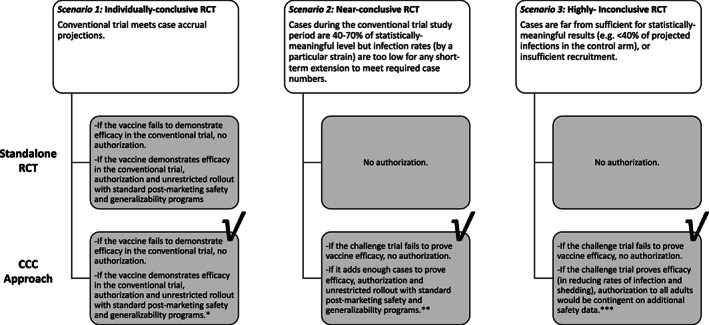
The only three possible scenarios on how conclusive the RCT is as a standalone in (upper dark box) a conventional RCT of 30 000 volunteers (randomized 1:1 between a SARS‐CoV‐2 vaccine and control), compared to [lower dark box] a CCC approach that combines such an RCT with a challenge trial (the latter also randomized 1:1 between the same vaccine and control). This perspective argues that under all three possible scenarios, the latter outcome is preferable, as indicated by the “check” sign, and explained in the following three notes. *Notes*: * Here, the challenge trial provides additional efficacy and safety data that increase confidence and potentially enable vaccine comparison, as well as other valuable information on, for example, correlates of protection. ** Here, the challenge trial “tops up” proof of efficacy on infection and shedding and provides additional valuable information, for example, on correlates of protection. The conventional trial provides safety data on 15 000 vaccinated volunteers. *** Here, the challenge trial provides proof of efficacy on infection and shedding.^8^, ^19^ Because data are insufficient to rule out widespread severity enhancement (there are too few subjects with exposure to vaccine+virus), additional safety data are required. If found safe, the vaccine can be authorized

Challenge trials could help reach the necessary case numbers—for example, by complementing conventional trials that assess vaccine impact on infection and infectiousness rates, with data on that impact from the challenge trial. That combination should be possible so long as the strain used in the challenge is relevant (e.g., it is the same strain as the one against which the trialists would like to assess vaccine efficacy in theconventional trial). On that assessment of the vaccine, a conventional trial of a SARS‐CoV‐2 vaccine candidate could be: 1) individually conclusive; 2) nearly individually conclusive, say, because the hotspot migrated elsewhere during the trial and starting from scratch in new sites would create unacceptable delay; or 3) far from individually conclusive, say, because the new availability of vaccines to the general population around sites thwarts further recruitment. What follows describes each scenario and explains why a CCC would work out better than a conventional trial (“RCT”) alone under that scenario. Figure 1 recaps these suggestions.

## 4 SCENARIO 1: A CONCLUSIVE CONVENTIONAL TRIAL

If the conventional trial shows the vaccine to be safe and efficacious, the authorization process will proceed as it normally does after a successful conventional trial, followed by broad distribution to the population and then, standard postmarketing safety requirements.^34^ In this happy scenario, though the challenge trial will not contribute any information strictly required for proving superiority to placebo, it would increase confidence and provide additional valuable information—on infection and shedding ratios for the relevant variant and regimen, and on the correlates of vaccine protection.

## 5 SCENARIO 2: MODEST CASE UNDER‐ACCRUAL IN THE CONVENTIONAL TRIAL

Suppose instead that the conventional trial recruits as planned. However, within an acceptable timeframe, it ascertains only 40–70% of the outcomes needed for statistical proof of efficacy on blocking infections against a particular strain. It therefore narrowly fails to produce evidence of a statistically significant benefit of the vaccine against that strain. That could come about in the ways noted above. First, with declining spread around the main sites, prolonging a conventional trial in the same site or recruiting in wholly new sites will sometimes still be unlikely to reach target numbers of outcomes within an acceptable duration. Second, in a waning COVID pandemic or in a future emerging infection outbreak with lower spread, there might not be the possibility of completing the trial by waiting longer or by moving to another site. Third, unless data collection stops based on the number of infections from a particular strain, only when data are analyzed would it become possible to tell whether exposures to a particular viral strain are sufficient for statistically meaningful proof of efficacy against that strain, or not.

In such circumstances, the combined data of both the conventional trial and a challenge trial (with the latter focused on a single strain of interest) could still be leveraged to rescue the vaccine while allowing timely authorization and dissemination. In this scenario, the result of the challenge trial component would provide the added proof of efficacy in reducing infection and shedding rates, which the inconclusive conventional trial individually failed to provide within an acceptable timeframe. Additionally, compared to a challenge trial only, the conventional trial would more than double the number of cases in which disease severity enhancement^31^ can either take place or not during the trial, clarifying that risk as well.

## 6 SCENARIO 3: SUBSTANTIAL CASE UNDER‐ACCRUAL IN THE CONVENTIONAL TRIAL, OR INCOMPLETE RECRUITMENT

If in the conventional trial, a waning pandemic makes cases fall far short of sufficiency (e.g., <40% of projected cases in the control arm) and sufficiency cannot be reached within an acceptable timeframe; or trialists cannot recruit enough participants once authorized vaccines become widely available around the site, the challenge trial would still provide proof of efficacy in reducing infection and shedding ratios, as under Scenario 2. However, the combined data alone would probably remain insufficient to support immediate authorization for all adults, due to insufficient data from the conventional trial to rule out severity enhancement in high‐risk adult populations excluded from the challenge trial. Before authorization for all adults can proceed, additional safety data would remain necessary. Gathering those data could take different forms.^8^, ^19^ As before, the challenge trial would generate additional useful data, for example, on correlates of protection.

## 7 IS CCC ETHICAL?

Some of us have elsewhere defended at length the ethics of vaccine efficacy testing in emerging infection outbreaks, through either conventional field trials^35^, ^36^ or human challenge trials.^8^, ^15^, ^18^, ^19^ We showed that either can be sufficiently consensual, tolerably safe, and so forth. But does an ethical problem arise from the combination of these two individually permissible designs, in the parallel approach? Opponents of such a combination might argue, first, that while each individual trial design has enough social value to justify its risks to individual participants when the alternative is no testing, the marginal social value of shifting from one trial (‐design) to two is too small to justify risks to the second trial's individual participants.

We believe the marginal social value of CCC against something like COVID will almost always remain sufficient. The added social value of surer, earlier, and more informative completion of testing of the central weapon against a pandemic which threatens an exceptional number of patients globally tends to be exceptionally high.^37^ That should typically keep the balance between that (exceptional) humanitarian value and the risks to individual participants (already accepted as tolerable in a single trial) highly favorable.

Another potential response by ethicist opponents of CCC might be that, by augmenting the combined number of participants (because two designs means at least two trials), CCC augments the overall chance of severe adverse events. Does that represent an ethical problem in CCC? We believe it does not, either.

It is true that more participants means greater chance of a severe adverse event in the combined set of participants. But research ethics is not primarily about protecting such combined social sets. It is mainly about protecting each individual participant, and that risk is not made worse by the existence of additional participants.^38^ For example, scholarship on when a medical trial's (net‐) risks are excessive (either compared to the trial's social benefits^39^, ^40^ or in absolute terms)^41^, ^42^, ^43^, ^44^ tends to focus on the risks to individuals participating, and not to the entire participant cohort. That is also in line with research ethics' classical individualist tenor, which pit collective utility against individual rights.^45^, ^46^ Indeed, if risk to social sets mattered more than protecting individuals in medical research, then surely the prevention of thousands or millions of deaths from stunted pandemic response would matter enough to justify CCC.

It may seem ethically preferable to prepare the challenge trial but to launch it only depending on whether the conventional trial yields inadequate results, instead of committing to holding a challenge trial. But this would either leave us with substantially less information on a pandemic risk or take months longer than launching the challenge earlier. In a pandemic, the value of obtaining information as early as possible is so vast that CCC is ethically preferable to any single trial, and preparations for a future pandemic should include all logistical and regulatory/oversight groundwork for a CCC.

## 8 CONCLUSION

So long as combining the two designs introduces no substantial delay, CCC improves vaccine development. CCC is the best choice for testing both authorized and next‐generation SARS‐CoV‐2 vaccines for new outcomes of interest and would make sense for some future outbreaks and pandemics. If at all affordable, funders and investigators should usually plan to perform both trial types, and lay the groundwork for both.

## CONFLICT OF INTEREST

The authors have no financial conflict of interests to declare. NE serves on the Advisory Board of challenge trial volunteer organization 1DaySooner, an unpaid position.
